# Supplementary material for: Greater transferability and accuracy of norm-conserving pseudopotentials using nonlinear core corrections
Source: Chem Sci. 2023 Sep 14;14(39):10934–43. doi: 10.1039/d3sc03709f (PMC10566506; doi:10.1039/d3sc03709f)
Supplement: SC-014-D3SC03709F-s001 [file SC-014-D3SC03709F-s001.pdf]

Electronic Supplementary Material (ESI) for Chemical Science.
This journal is © The Royal Society of Chemistry 2023

Supplementary Information: Greater Transferability and Accuracy of Norm-conserving Pseudopotentials using Nonlinear Core Corrections

Wan-Lu Li[1−3], Kaixuan Chen[1−3], Elliot Rossomme[1,2], Martin Head-Gordon[1−3], Teresa Head-Gordon[1−5*]

[1]*Kenneth S. Pitzer Center for Theoretical Chemistry,* [2]*Department of Chemistry,* [3]*Chemical Sciences Division, Lawrence Berkeley National Laboratory,* [4]*Department of Chemical and Biomolecular Engineering,* [5]*Department of Bioengineering, University of California, Berkeley, Berkeley, California 94720, USA*

E-mail: thg@berkeley.edu

Table S1. Electronic configurations of core and valence regions for pseudopotentials of elements studied.

Element	Functional	NLCC	Core	Valence
H, Li, Be	PBE	N	None	All
	PBE0	N	None	All
	ωB97M-rV	N	None	All
	B97M-rV	N	None	All
B, C, N, O, F	PBE	Y	[He]	$2s^22p^x$
	PBE0	Y	[He]	$2s^22p^x$
	ωB97M-rV	Y	[He]	$2s^22p^x$
	B97M-rV	Y	[He]	$2s^22p^x$
Na, Mg	PBE	N	[Ne]	$3s^23p^x$
	PBE0	N	[Ne]	$3s^23p^x$
	ωB97M-rV	N	[Ne]	$3s^23p^x$
	B97M-rV[a]	N	[Ne]	$3s^23p^x$
Al, Si, P, S, Cl	PBE	Y	[Ne]	$3s^23p^x$
	PBE0	Y	[Ne]	$3s^23p^x$
	ωB97M-rV	Y	[Ne]	$3s^23p^x$
	B97M-rV[a]	N	[Ne]	$3s^23p^x$

[a] At the B97M-rV level, we initially tried to optimize the third-row elements. However, due to substantial errors, we were unable to obtain reliable results. Therefore, we resorted to using the GTH/MOLOPT method without B97M-rV to generate accurate results for these heavier elements.

Table S2. Comparison of mean absolute deviations (kcal/mol) for a specific atom type within the G2 dataset.[1–3] Predictions were made at the PBE and B97M-rV levels using the complete basis set def2-TZVPPD, with and without NLCC correction.

Functional	Pseudopotential/Basis	B	C	N	O	F
PBE	GTH/def2-TZVPPD	12.76	20.52	22.19	19.51	16.58
	GTH-NLCC-2013/def2-TZVPPD	2.44	2.19	1.56	1.28	9.13
B97M-rV	GTH/def2-TZVPPD	29.90	29.42	38.98	24.47	18.25
	GTH-NLCC-OPT-all[a]/def2-TZVPPD	27.55	11.28	10.79	11.14	9.00
	GTH-NLCC-OPT[b]/def2-TZVPPD	4.07	6.43	10.70	6.68	8.06
Functional	Pseudopotential/Basis	Al	Si	P	S	Cl
PBE	GTH/def2-TZVPPD	4.11	2.83	2.91	9.40	7.71
	GTH-NLCC-2013/def2-TZVPPD	8.34	2.23	1.30	1.21	6.62
B97M-rV	GTH/def2-TZVPPD	4.22	5.96	7.85	11.38	10.19
	GTH-NLCC-OPT-all[a]/def2-TZVPPD	25.61	10.26	9.03	30.51	18.92
	GTH-NLCC-OPT[b]/def2-TZVPPD	2.86	5.10	7.17	4.17	2.80

[a] GTH-NLCC-OPT-all means that NLCC parameters are optimized for all elements including the second and third rows.[b] GTH-NLCC-OPT means that NLCC parameters are optimized only for the second-row elements.

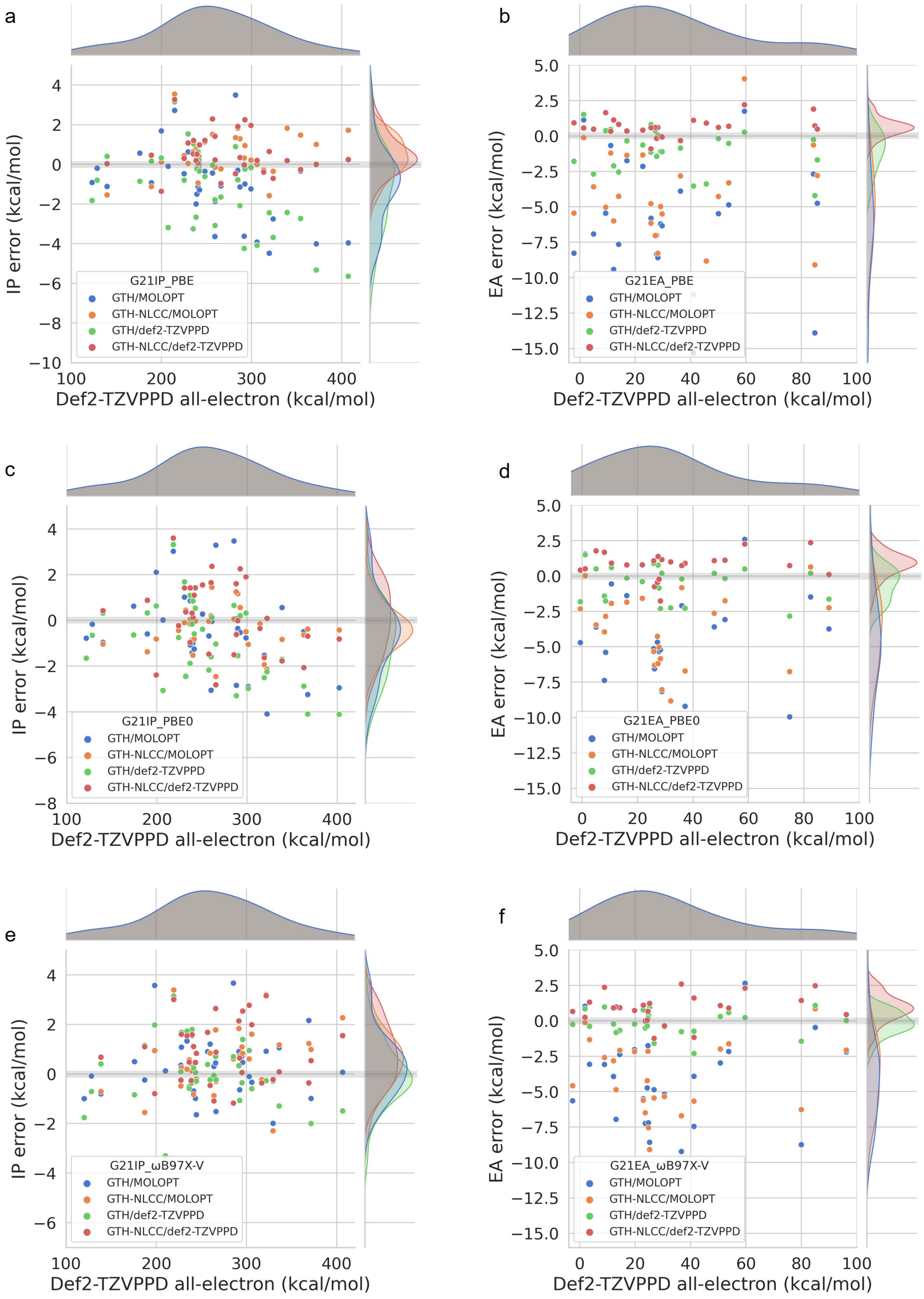

Figure S1: Distributions of MADs and all-electron calculations at the levels of PBE, PBE0 and ωB97X-V for IP and EA datasets.[4,5]

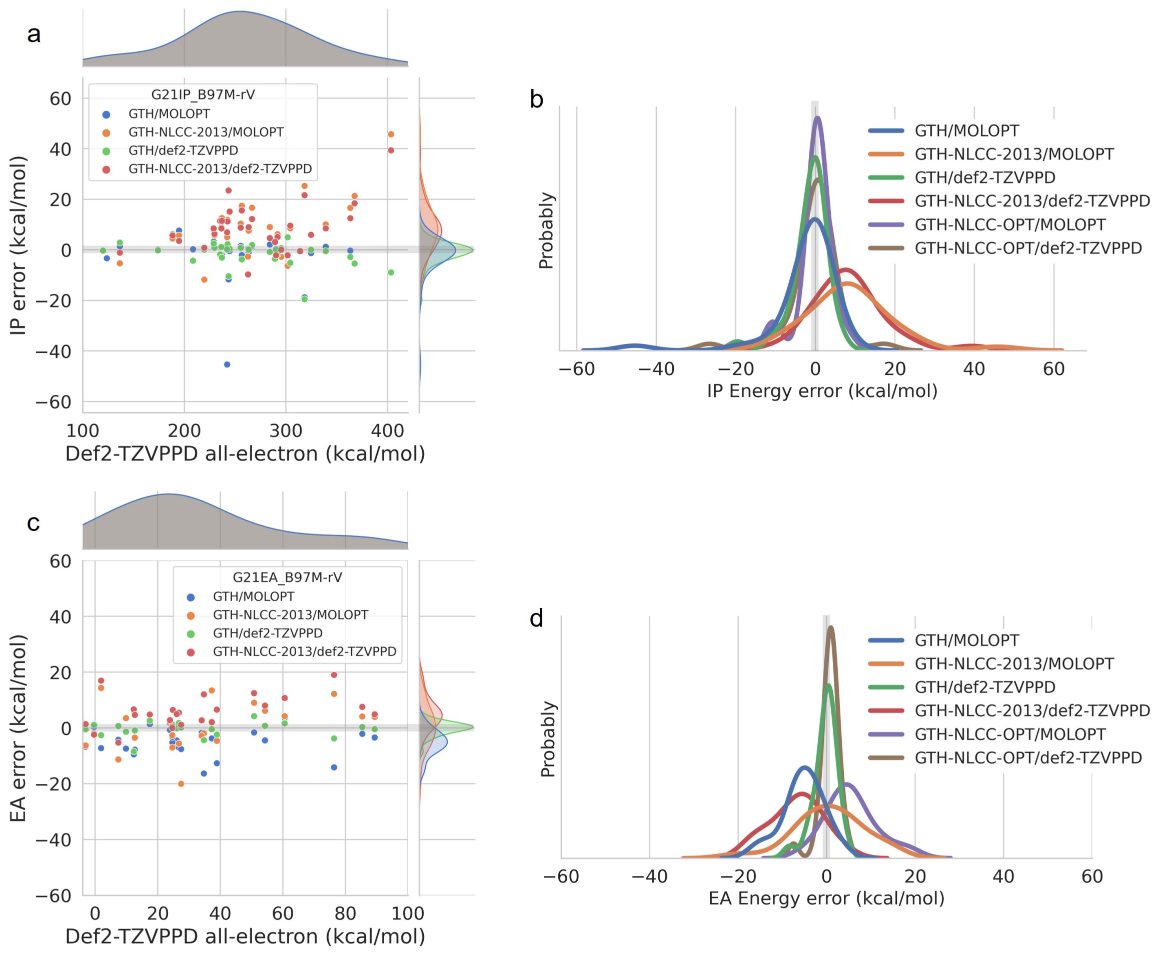

Figure S2: Distributions of MADs and all-electron calculations at the level of B97M-rV for IP and EA datasets.

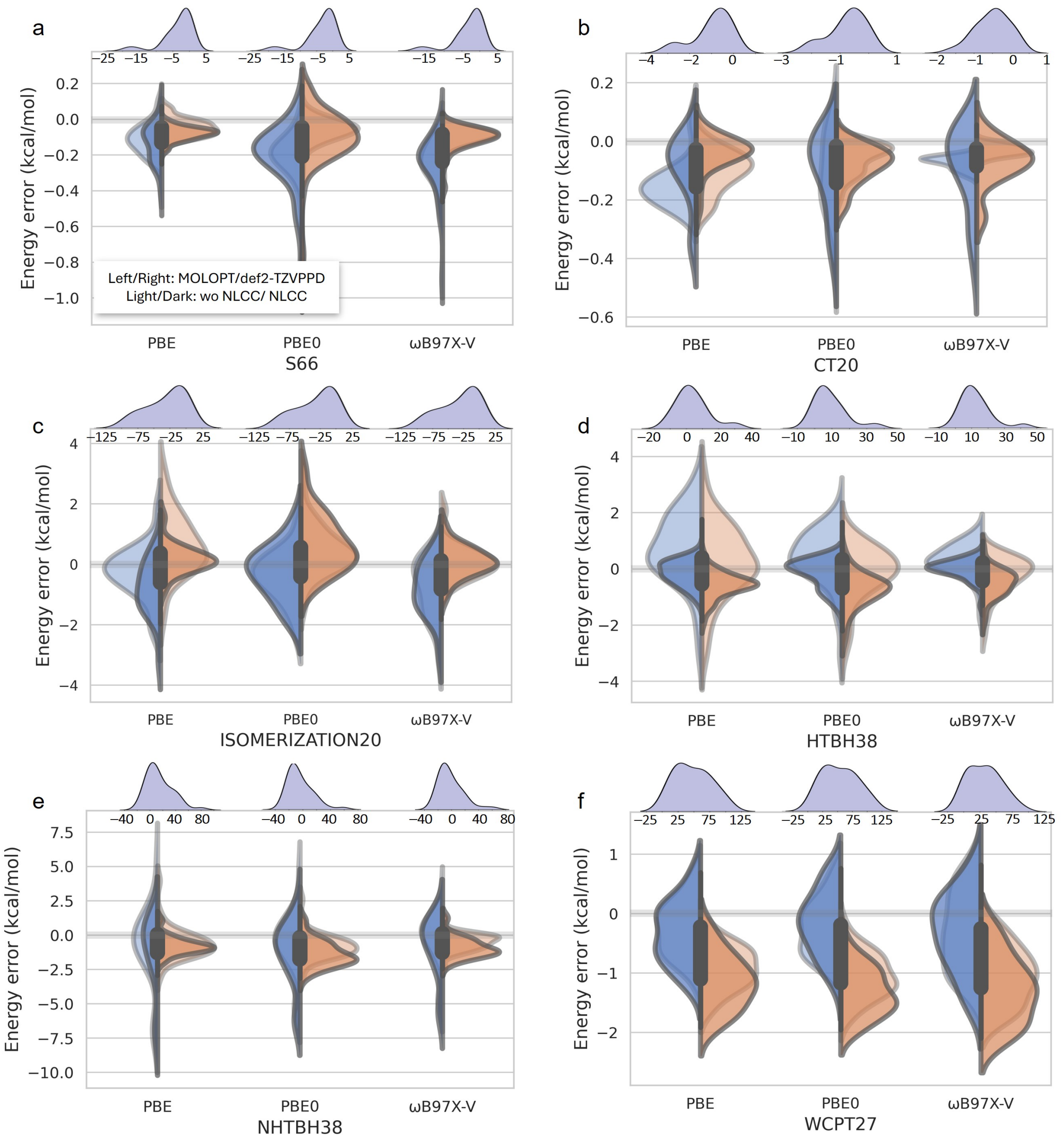

Figure S3: NLCC performance on non-thermochemical properties. The energy errors are compared with def2-TZVPPD all-electron calculations, of which the distribution and range are shown by the density plot on the top of each subplot.

References

(1) Curtiss, L. A.; Raghavachari, K.; Redfern, P. C.; Pople, J. A. Assessment of Gaussian-2 and density functional theories for the computation of enthalpies of formation. *J. Chem. Phys.* **1997**, *106*, 1063–1079.

(2) Pople, J. A.; Head-Gordon, M.; Fox, D. J.; Raghavachari, K.; Curtiss, L. A. Gaussian-1

theory: A general procedure for prediction of molecular energies. *J. Chem. Phys.* **1989**, *90*, 5622–5629.

(3) Curtiss, L. A.; Jones, C.; Trucks, G. W.; Raghavachari, K.; Pople, J. A. Gaussian-1 theory of molecular energies for second-row compounds. *J. Chem. Phys.* **1990**, *93*, 2537–2545.

(4) Curtiss, L. A.; Raghavachari, K.; Trucks, G. W.; Pople, J. A. Gaussian-2 theory for molecular energies of first- and second-row compounds. *J. Chem. Phys.* **1991**, *94*, 7221–7230.

(5) Goerigk, L.; Grimme, S. Efficient and Accurate Double-Hybrid-Meta-GGA Density Functionals—Evaluation with the Extended GMTKN30 Database for General Main Group Thermochemistry, Kinetics, and Noncovalent Interactions. *J. Chem. Theory Comput.* **2011**, *7*, 291–309.
